# Comprehensive proximity proteomics expand the known interactome of the oncoprotein β-catenin

**DOI:** 10.1038/s41598-026-68008-5

**Published:** 2026-09-01

**Authors:** Lavanya Moparthi, Wenjing Zhong, Stefan Koch

**Affiliations:** 1 Department of Biomedical and Clinical Sciences (BKV), BKV/MMV, Linköping University, Plan 13, Lab 1, 58185 Linköping, Sweden; 2 Wallenberg Centre for Molecular Medicine (WCMM), Linköping University, Linköping, Sweden

**Keywords:** β-catenin, Proteomics, Mass spectrometry, BioID, TurboID, Biological techniques, Cell biology, Computational biology and bioinformatics, Molecular biology

## Abstract

The oncoprotein β-catenin has critical roles in cell adhesion and cell signalling. β-catenin affects human physiology and pathology through numerous interaction partners, of which many have been discovered by standard affinity purification-based proteomics. However, the interaction landscape of β-catenin remains incompletely understood, highlighting a need for new experimental approaches for the exploration of β-catenin biology. Proximity proteomics, which facilitate the discovery of molecules vicinal to proteins-of-interest by mass spectrometry, have recently emerged as a powerful complementary tool for the study of protein–protein interactions, but have not been applied to β-catenin so far. We investigated the interactome of β-catenin in model cell lines by proximity proteomics using expression constructs with the biotin ligases BioID and TurboID. Mass spectrometry analyses following biotin labelling identified numerous candidate interactors of β-catenin, including many that had not been observed in earlier studies using standard proteomics. Enrichment analyses suggested that proximity proteomics capture proteins associated with the known biological functions of β-catenin, including cell adhesion, Wnt/β-catenin signalling, and transcription regulation. The molecular tools and data generated in this study provide new insights into β-catenin biology and highlight potential new regulators of β-catenin function that warrant further exploration.

**Supplementary Information:**

The online version contains supplementary material available at https://doi.org/10.1038/s41598-026-68008-5.

## Introduction

Β-catenin is a highly conserved scaffold protein with critical functions in cell adhesion and cell signalling^1^. In the plasma membrane, β-catenin acts as an adapter molecule that connects E-cadherin to α-catenin in adherens junctions. Additionally, cytoplasmic β-catenin is an essential transcription co-factor of, among others, TCF/LEF family transcription factors in the Wnt/β-catenin signalling pathway. Wnt/β-catenin signalling promotes stem cell maintenance and turn-over and is limited in differentiated cells through the constitutive proteasomal degradation of β-catenin mediated by a multi-protein destruction complex. Spontaneous mutations that increase the stability of β-catenin are causatively involved in the pathogenesis of many types of cancer, especially colorectal cancer, in which most tumours are initiated by aberrant Wnt/β-catenin pathway activation^2,3^. Because of its prominent roles in human physiology and pathology, β-catenin has been studied extensively for several decades, and its function and regulation are generally well-understood. However, some aspects of its biology, such as the tissue-specific regulation of β-catenin-dependent gene transcription^4^ or the precise mode of its subcellular trafficking^5^, remain incompletely resolved and under active investigation.

Considering that β-catenin requires various interaction partners to exert its functions, affinity purification/mass spectrometry (AP-MS)-based proteomics assays have been crucial in identifying important β-catenin interactors in different contexts and continue facilitating the discovery of new interactors to this day^6–12^. In this study, we generated and tested expression constructs of β-catenin fused to BioID^13^ or its derivative TurboID^14^ for proximity labelling. BioID and TurboID, which has a substantially faster reaction time, are based on the *Escherichia coli* biotin ligase BirA that allows for stable labelling of vicinal proteins of proteins-of-interest in living cells upon addition of biotin. Biotin-labelled proteins can be easily isolated by streptavidin pull-down, thus enabling convenient analysis of interacting proteins by mass spectrometry (i.e., proximity proteomics). AP-MS and proximity proteomics are complementary methods that capture largely discrete yet biologically meaningful sets of interactors^15^, which means that proximity proteomics may increase our understanding of the interactome of β-catenin. Using this approach, we identify numerous known as well as new potential β-catenin interactors in 293 T cells, and highlight ABCF2 as a putative functional β-catenin interactor. We conclude that proximity proteomics are a useful tool for the further exploration of β-catenin biology.

## Materials and methods

### Cell culture and plasmid construction

Authenticated 293 T and HCT116 cells were obtained from the German Collection of Microorganisms and Cell Cultures (DSMZ, Braunschweig, Germany). DLD1 cells were a kind gift from Dr. Xiao-Feng Sun (Linköping University). The cells were cultured in DMEM supplemented with 10% fetal bovine serum, 2 mM glutamine, and 1% penicillin/streptomycin. Cells were maintained in a 5% CO_2_ incubator at 37 °C and routinely tested negative for mycoplasma contamination by analytical qPCR (Eurofins Genomics, Ebersberg, Germany). BioID and TurboID tagged β-catenin constructs were generated using standard enzymatic restriction digestion and ligation with T4 DNA ligase. Plasmids were validated by Sanger sequencing (Eurofins Genomics). Stable cell lines were generated by cloning TurboID in place of GFP in pMaCTag-P05 plasmid (Addgene plasmid # 120,016, a gift from Michael Knop) and performing gene editing using a CRISPR-Cas12a-based method^16^. After puromycin selection, clonal cell lines were derived by limiting dilution. All newly generated constructs have been submitted to the Addgene repository (Addgene plasmid # 242,995, 242,996, 242,998, 242,999). mCherry-Beta-Catenin-20 (mCherry-CTNNB1; Addgene plasmid # 55,001, a gift from Michael Davidson) was used for overexpression of wild-type β-catenin.

### Luciferase reporter assay

The dual luciferase assay was conducted as described previously^17^. Briefly, cells were seeded on a 96-well plate and transfected with 50 ng of TOPFlash reporter (Addgene plasmid # 12,456, a gift from Randall Moon^18^), 5 ng of Renilla luciferase control (Addgene plasmid # 12,179, a gift from David Bartel) and 10 ng of β-catenin plasmids. After 24 h of transfection, cells were lysed in a passive lysis buffer (25 mM Tris, 2 mM DTT, 2 mM EDTA, 10% (v/v) glycerol, 1% (v/v) Triton X-100, pH 7.8) by shaking for 10 min and then the lysates were transferred to a flat-bottomed 96-well luminescence assay plate. First, luciferin containing firefly luciferase buffer was added to the plate and luminescence was measured within 10 min using a SpectraMax iD3 Multi-Mode Microplate Reader (Molecular Devices). Next, coelenterazine-h (Promega) containing Renilla luciferase buffer was added to the plate and luminescence was measured immediately. Each experiment was performed in triplicate and data were normalised to the Renilla luminescence values.

### Antibodies and reagents

The following primary antibodies were used: rabbit anti-Histone H3 (PA5-16,183, Invitrogen), mouse anti-Flag M2 (F3165, Sigma Aldrich), mouse anti-β-catenin (sc-133240, Santa Cruz), rabbit anti-ABCF2 (10,226–1-1AP, Proteintech), rabbit anti-HSP70 (AF1663, R&D Systems), and rabbit anti-HA (NB600-363, Novus Biologicals). Near infrared (NIR) fluorophore-labelled secondary antibodies and IRDye 800CW-linked Streptavidin were purchased from LI-COR. Non-immune IgG control antibodies were from Invitrogen. Dicer-ready siRNAs (DsiRNA) against ABCF2 (hs.Ri.ABCF2.13.1 and hs.Ri.ABCF2.13.2) were purchased from Integrated DNA Technologies. DAPI (4’,6-diamidino-2-phenylindole) was obtained from Thermo Fisher Scientific.

### Immunoblotting and immunoprecipitation

The cells were harvested in PBS and lysed with 1% NP40 in TBS supplemented with a 1 × Complete protease inhibitor cocktail (Pierce) on ice for 10 min. Then, the lysates were sonicated and centrifuged at 13,000 rpm for 10 min to remove cell debris. Subcellular fractionation was performed with the REAP method^19^. Protein lysates were boiled with 4 × Laemmli sample buffer with 50 mM DTT, separated on 10% polyacrylamide gel, and transferred to nitrocellulose membranes. The membrane was incubated in a blocking buffer for 1 h and blotted with primary antibodies. The primary antibodies were detected by Near-infrared (NIR)-labelled secondary antibodies. Blots were visualised using the LI-COR CLx imaging system (LI-COR) controlled through ImageStudio software.

For immunoprecipitation, DLD1 whole cell lysates were pre-cleared with Protein A/G Plus agarose beads (Santa Cruz) for 1 h at 4 °C, and incubated with 2 µg primary antibodies overnight at 4 °C with end-over-end rotation. Following repeated washes with TBS/0.1% Tween-20, the beads were boiled in 4 × Laemmli sample buffer with 50 mM DTT.

Uncropped images of all immunoblots presented in this manuscript can be found in the supplemental information.

### Immunofluorescence microscopy

HEK293T cells and endogenously CTNNB1-TurboID-tagged cells were incubated for 21 h and then treated with or without 500 µM Biotin for 3 h. Cells were fixed with 4% w/v paraformaldehyde for 15 min, then permeabilised with 0.25% v/v Triton X-100 for 5 min and blocked with 5% w/v bovine serum albumin for 1 h. The cells were incubated with rabbit anti-HA for the detection of TurboID-tagged CTNNB1. The cells were then washed and incubated with anti-rabbit DY488 secondary antibody for the detection of the primary antibody. For the detection of biotinylated proteins, Alexa Fluor 488-conjugated streptavidin antibody (S11223, Thermo Fisher Scientific) was used. Cells were mounted with ProLong Glass Antifade Mountant with NucBlue (Invitrogen). Images were acquired using an LSM700 confocal laser scanning microscope controlled through ZEN software (Carl Zeiss, Jena, Germany), and post-processed using ImageJ 1.52p (NIH, Bethesda, USA).

### Proximity labelling mass spectrometry

The BioID and TurboID samples for mass spectrometry were performed basically as described previously^13,14,20,21^. Briefly, BioID- and TurboID-tagged β-catenin and control plasmids were transfected into 293 T cells using Lipofectamine 2000 (Thermo Fisher Scientific). For BioID labelling, after 6 h of transfection, cells were treated with 50 μM biotin, and incubated for 18 h at 37 °C, 5% CO_2_. For TurboID, cells were treated with 500 μM biotin after 21 h of transfection and incubated for 30 min and 3 h at 37 °C, 5% CO2. For endogenous beta-catenin TurboID experiments, a successfully tagged clone was cultured for 21 h and then incubated with 500 µM biotin for 3 h at 37 °C, 5% CO2. The cells were surface washed with PBS before harvesting and cell pellets were washed three times with PBS to remove any remaining biotin.

For BioID, cells were lysed in RIPA buffer (Thermo Fisher Scientific) for 1 h at 4 °C with end-over-end rotation, then sonicated and centrifuged at 13,000 rpm for 30 min at 4 °C. Pre-washed streptavidin beads (GE Healthcare, USA) were added to the cell lysate and incubated for 3 h at 4 °C with end-over-end rotation. The beads were collected by centrifuging at 2000 rpm for 2 min and then washed four times with 50 mM ammonium bicarbonate (NH_4_HCO_3_). On-bead digestion was performed by adding 100 μl of freshly prepared 1 µg of trypsin (Thermo Fisher Scientific) in 50 mM NH_4_HCO_3_ to the beads followed by overnight at 37 °C with end-over-end rotation.

For proximity labelling by TurboID, cells were lysed in RIPA buffer containing 1 × Complete protease inhibitor cocktail for 5 min on ice. Then the samples were sonicated, and cell debris was removed by centrifuging at 10,000 rpm for 10 min. Prewashed streptavidin beads (GE Healthcare) were added to the cell lysate and incubated for 1 h at RT then overnight at 4 °C with end-over-end rotation. The beads were washed twice with 1 ml of RIPA buffer, once with 1 ml of 1 M KCl, once with 1 ml of 0.1 M Na2CO3, once with 1 ml of 2 M urea in 50 mM Tris–HCl, pH 7.5, and twice with 1 ml RIPA lysis buffer. The beads were then transferred to a new Eppendorf tube and washed twice with 50 mM Tris–HCl buffer, pH 7.5 and 2 M urea/50 mM Tris–HCl, pH 7.5 buffer. Beads were incubated with 0.4 μg of trypsin (Thermo Fisher Scientific) in 2 M urea/50 mM Tris–HCl, pH 7.5,1 mM DTT buffer for 1 h at RT with end-over-end rotation. After incubation, the supernatant was collected, and the beads were washed twice with 60 μl of 2 M urea/50 mM Tris–HCl buffer, pH 7.5 and the washes were combined with the collected supernatant. The supernatant was first reduced with 4 mM DTT for 30 min at RT, then alkylated with 10 mM iodoacetamide for 45 min in the dark at RT with end-over-end rotation. An additional 0.5 μg of trypsin was added to allow the complete digestion of the samples and incubated overnight under the same conditions. The digested samples were desalted with Pierce C18 pipette tips (Thermo Fisher Scientific) and then dried with a vacuum centrifuge.

BioID and samples were analysed by mass spectrometry, using an Easy nano LC II HPLC interfaced with a nanoEasy spray ion source (Thermo Fisher) connected to an LTQ Orbitrap Velos Pro hybrid mass spectrometer (Thermo Fisher). The peptides were loaded on an NS-MP-10 Biosphere C18 column, 2 cm (100 μm inner diameter, packed with 5 μm resin), and the chromatographic separation was performed at RT on a 10.1 cm (75 μm inner diameter) NS-AC-10-C18 column packed with 5 μm of resin (NanoSeperations, Netherlands). The nanoHPLC was operating at 300 nl/min flow rate with a linear gradient of solvent B (0.1% (v/v) formic acid in acetonitrile) in solvent A (0.1% (v/v) formic acid in water) for 60 min. Full MS scans (380–2000 m/z) were recorded at a resolution of 30,000. The top 20 most intense multiple charged ions were selected with an isolation window of 2.0 and fragmented in the linear ion trap by Collision-induced dissociation.

TurboID-labelled samples and endogenous TurboID-tagged samples were analysed as previously described^20^ using an EASY nLC 1200 system interfaced with a nanoEasy spray ion source (Thermo Scientific) connected to a Q Exactive HF Hybrid Quadrupole-Orbitrap Mass Spectrometer (Thermo Scientific).

Raw data were processed by Proteome Discover 2.0 (Thermo Fisher Scientific) searching against the Homo sapiens UniProt database with the Sequest HT search engine. The search parameters were as follows: enzymes: trypsin with two missed cleavages, no variable modifications (BioID and TurboID data); fixed modification: Carbamidomethylation (TurboID data); Peptide Mass Tolerance, 10 ppm; MS/MS Fragment Tolerance, 0.5 Da (BioID data), 0.02 Da (TurboID data). Quantification of the analyzed data was performed using Scaffold 5.1.0 (Proteome Software Inc, https://www.proteomesoftware.com/products/scaffold-5) using total spectral counts. Protein and peptide identifications were accepted if they could be established at greater than 95 and 90% probability, respectively and if protein identification contained at least two identified peptides.

### Proximity ligation assay (PLA)

PLA was performed using a commercially available NaveniBright—MR, HRP kit (Navinci Diagnostics) according to the manufacturer’s instructions. Briefly, cells were fixed, permeabilized, and incubated with primary antibodies against β-catenin and ABCF2, followed by incubation with species-specific PLA probes. Ligation and amplification reactions were carried out to generate detectable signals. The PLA signal was visualized through an HRP-mediated chromogenic reaction that produces a brown precipitate at sites of protein proximity. Nuclear staining was performed using the NaveniBright nuclear stain, resulting in a blue counterstain.

PLA images were acquired using an Olympus BX51 microscope equipped with an Olympus XC30 camera and cellSens software. Brightfield images were captured using a 60 × oil-immersion objective. Exposure was controlled automatically by the acquisition software. Images were acquired at a resolution of 2080 × 1544 in full-frame mode. All images were acquired using identical acquisition settings across all experimental groups to ensure comparability and represent single focal planes. Multiple fields of view were examined for each condition. No post-acquisition adjustments were applied.

### Data analysis

Proteomics data were processed in R v4.4.0 essentially as described previously^20^. BioID and TurboID data were further analysed in SAINTexpress^22^ v3.6.3, where significance was assumed at a Bayes false discovery rate (BFDR) of < 0.05 and SAINT score ≥ 0.65, as reported previously^23^. SAINTexpress analyses were performed on the complete datasets, without prior removal of possible contaminants. For data harmonisation and cross-comparison, spectral counts were first normalised by subtracting the corresponding averaged control counts. Then, values were uniformly scaled using the rescale function in R package scales v1.4. Enrichment analyses were performed using clusterProfiler v4.12.6^24^, which uses hypergeometric testing to determine enrichment. Tree plots were generated using enrichplot v1.30.5, based on the Jaccard similarity coefficient of the most enriched gene ontology terms. Possible contaminants in the proteomics data were classified using the CRAPome database^25^. Specifically, we used a cutoff of detection frequency (number of experiments in which a protein was detected divided by the total number of experiments) of ≥ 0.2 and average spectral count of ≥ 3 in the CRAPome database “H. sapiens – Proximity Dependent Biotinylation” (accessed in November 2025 at https://reprint-apms.org/?q=viewproteinprofiles). R square values were derived from Pearson’s product-moment correlation estimate.

## Results

### Construction and validation of BioID and TurboID fused to β-catenin

**Fig. 1:**
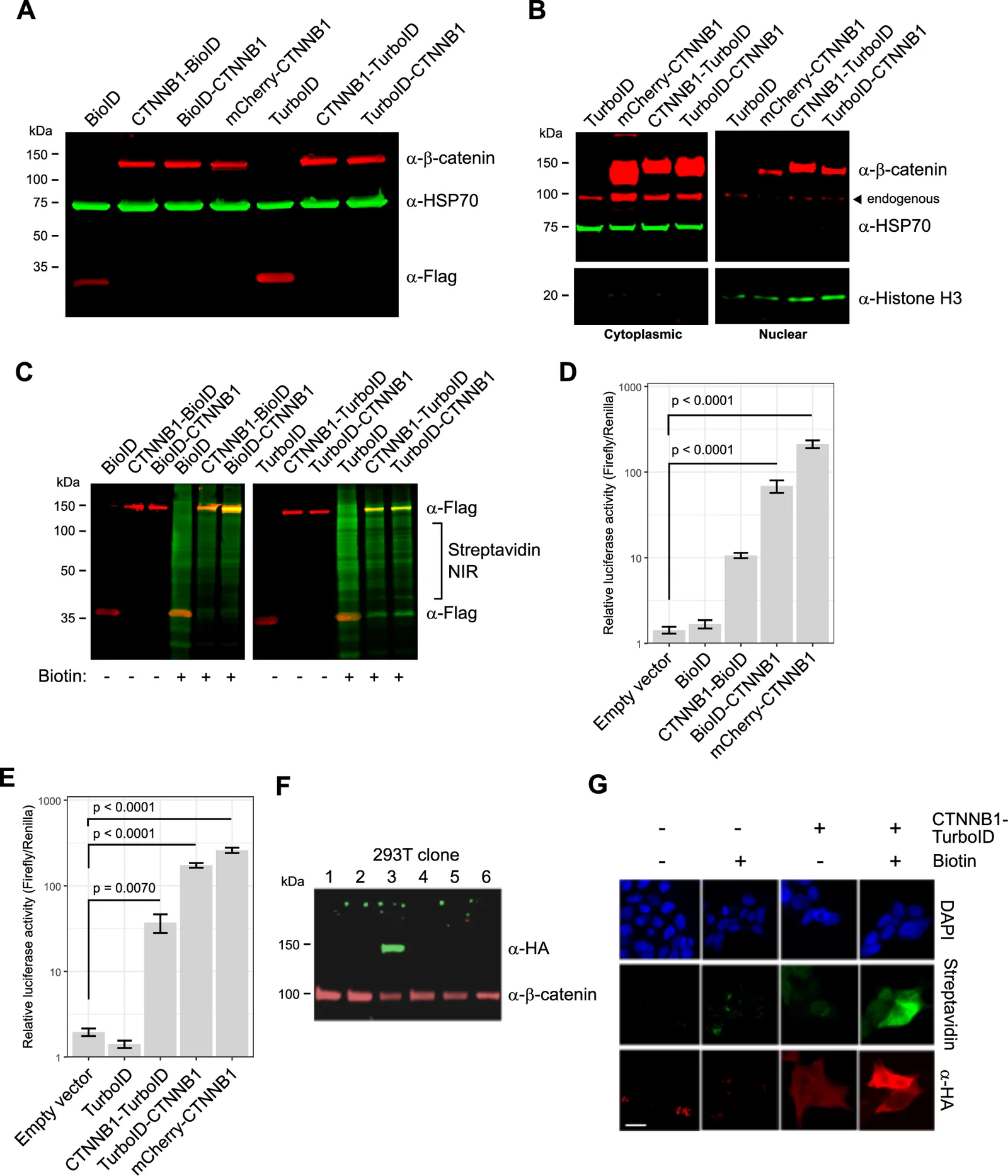
Construction and validation of β-catenin constructs for proximity proteomics. (**A**) Immunoblot of β-catenin BioID and TurboID constructs expressed in 293 T cells. HSP70 was used as a loading control. (**B**) Immunoblot of β-catenin TurboID constructs expressed in 293 T cells, following subcellular fractionation. HSP70 and Histone H3 were used as loading controls. (**C**) Immunoblot of β-catenin BioID and TurboID constructs expressed in 293 T cells. Where indicated, cells were treated with biotin for 18 h. Biotinylated proteins were detected using near-infrared (NIR)-labelled streptavidin. (**D, E**) TOPflash reporter assays of β-catenin-dependent transcriptional activity in 293 T cells. Data were analysed using Dunnett’s post-hoc test following one-way ANOVA. n = 3 biological replicates per condition. (**F**) Immunoblot of endogenously tagged CTNNB1-TurboID in 293 T single cell clones. (**G**) Immunocytochemistry of endogenous CTNNB1-TurboID (HA, red) and biotinylated proteins (streptavidin, green) in 293 T clonal cell lines. Abundant protein biotinylation can be observed following biotin treatment. Nuclei were counterstained with DAPI (blue). Scale bar: 10 µm.

We generated tagged expression constructs of β-catenin by restriction cloning of full-length cDNA of human β-catenin, encoded by *CTNNB1*, into a pCS2 + plasmid with N or C-terminal BioID or TurboID containing a Flag epitope tag. Immunoblotting confirmed expression of the constructs at the expected molecular weight (Fig. 1A). Subcellular fractionation of 293 T cells expressing the TurboID constructs additionally confirmed that these proteins localised to both the cytoplasm and nucleus, and did not affect the abundance of endogenous β-catenin (Fig. 1B). Biotin treatment resulted in comparable, uniform protein biotinylation across all constructs (Fig. 1C). Additionally, we validated the function of our constructs using the β-catenin/TCF reporter plasmid TOPflash, which encodes luciferase under the control of multiple TCF binding sites^18^. All constructs activated the TOPflash reporter, which confirms functional activity of the β-catenin fusion constructs, albeit to a lesser extent than wild-type β-catenin (Fig. 1D, E). This loss off activity, especially in the C-terminal fusion constructs, is expected due to likely interference of the BioID/TurboID tags with the transactivation domains of β-catenin^26,27^, but should not affect their usefulness for proximity labelling. Lastly, we generated 293 T cell lines with endogenously tagged β-catenin by CRISPR-Cas12a-based gene editing^16^ with an HA-tagged TurboID template. Following puromycin selection, we derived clonal cell lines by limiting dilution. Construct expression was confirmed by immunoblotting, as before (Fig. 1F). Moreover, using immunofluorescence microscopy, we observed extensive streptavidin labelling in cells after addition of biotin (Fig. 1G). In summary, we have generated and validated a toolkit of β-catenin fusion constructs for biotin labelling of vicinal proteins. All plasmids described here have been deposited in the Addgene plasmid repository.

### Exploration of the β-catenin interactome using BioID

**Fig. 2:**
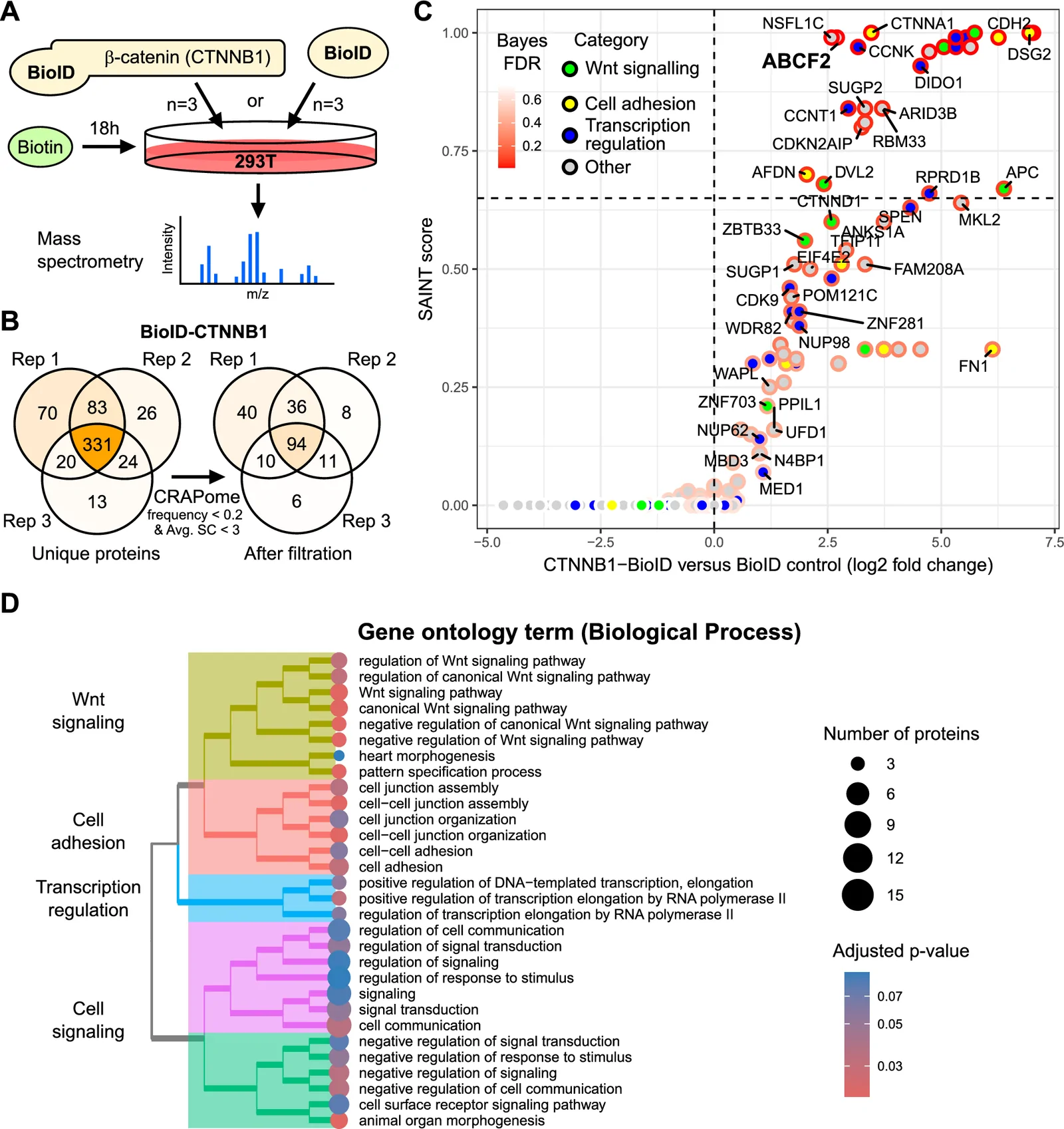
Mapping of the β-catenin proximity interactome using BioID. (**A**) Schematic workflow of the experiment. (**B**) Venn diagrams of putative β-catenin interactors identified in the individual experiments (Reps 1–3) before (left) and after removal of possible contaminants using the CRAPome repository (right). SC: spectral count. (**C**) SAINTexpress analysis of the combined BioID results. Categories of candidate β-catenin interactors were derived from their primary gene ontology annotations. Possible contaminants, defined as detection frequency of ≥ 0.2 and average SC of ≥ 3 in the CRAPome database, were removed prior to plotting and subsequent gene ontology analysis. FDR: false discovery rate. (**D**) Gene ontology analysis of candidate β-catenin interactors identified by BioID (SAINT score > 0.65 and Bayes FDR < 0.05).

We first explored the proximity interactome of β-catenin using the BioID-based construct, BioID-CTNNB1, which we transiently overexpressed in 293 T cells (Fig. 2A). Following biotin labelling for 18 h and streptavidin pull-down, we identified 748 unique proteins by mass spectrometry across all assays, including controls (Supplemental Table 1). After the removal of likely contaminants, i.e., proteins that are commonly detected in proteomics assays and are therefore potential false-positives^25^, this number dropped to 324 proteins, possibly suggesting that proximity biotinylation frequently detects high abundance proteins. Among proteins identified in the BioID-CTNNB1 samples, 73% were found in at least two biological replicates (Fig. 2B), indicating high reproducibility of the findings. Subsequent data analysis using SAINTexpress^22^ highlighted 34 proteins that were significantly enriched compared to BioID control (Fig. 2C, and Supplemental Table 2). This list contained several known interactors of β-catenin in cell junctions (e.g., α-catenin, γ-catenin, and N-cadherin) and the Wnt/β-catenin signalling pathway (e.g., APC, ICAT, and DVL-2), thus supporting the validity of the data. Gene ontology analysis of the enriched proteins showed that these proteins are mainly involved in cell signalling, cell adhesion, and transcription regulation (Fig. 2D), which agrees with the well-established functions of β-catenin.

### Further exploration of the β-catenin interactome using TurboID

**Fig. 3:**
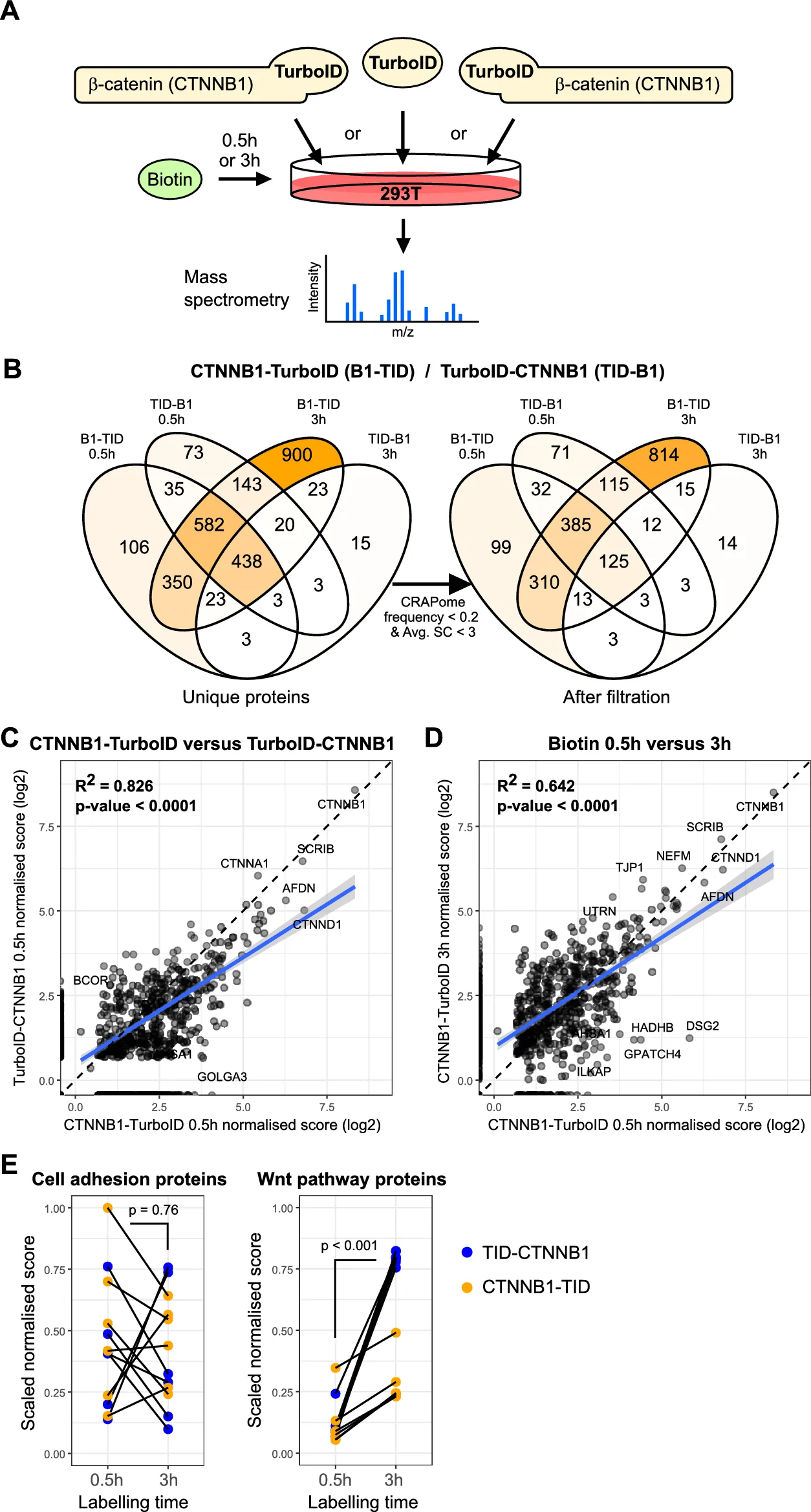
Mapping of the β-catenin proximity interactome using TurboID. (**A**) Schematic workflow of the experiment. (**B**) Venn diagrams of putative β-catenin interactors identified in the individual experiments before (left) and after removal of possible contaminants using the CRAPome repository (right). SC: spectral count. (**C**, **D**) Scatter plot of hits identified dependent on the position of the TurboID domain (**C**) or the biotin labelling time (**D**). Data were normalised to the corresponding control condition and analysed using Spearman’s rank correlation. (**E**) Changes in the normalised spectral counts of cell adhesion (left: AFDN, CTNNA1, CTNND1, SCRB, DSG2, CDH2, TJP1) and Wnt pathway-associated proteins (right: GSK3A, GSK3B, DVL2, DVL3, AXIN1, APC, BTRC) dependent on the biotin labelling time. Data were normalised to the respective control conditions, scaled for easier comparison, and analysed using Welch’s paired t-test.

Having established that proximity proteomics can facilitate the discovery of β-catenin interactors, we next repeated proximity labelling using the β-catenin constructs with N or C-terminally fused TurboID (Fig. 3A). Since TurboID allows for considerably shorter labelling times than BioID, we tested biotin treatment for both 0.5 h and 3 h. Across all assays, including controls, we identified 3,287 unique proteins by mass spectrometry, of which 2,524 remained after removal of likely contaminants (Supplemental Table 3). While this number was substantially greater than results from BioID experiments, we noticed lower consistency across individual assays, with 51% of proteins that were found in two or more experiments (Fig. 3B). We then investigated if the location of the TurboID domain and biotin labelling time affect protein discovery (Fig. 3C, D). After subtraction of the corresponding control condition, we observed that similar proteins were discovered irrespective of the location of the TurboID domain (Fig. 3C). Similarly, proteins that were identified after both 0.5 h or 3 h of biotin labelling displayed very comparable spectral counts (Fig. 3D). The interacting proteins identified in all conditions with the highest normalised spectral counts included several structural proteins that are known interactors of β-catenin in adherens junctions (e.g., α/δ-catenin, scribble, afadin), suggesting that proximity biotinylation may preferentially identify interactors of plasma membrane-associated β-catenin. To explore these results further, we focused on a selection of well-established β-catenin interactors in cell adhesions and the Wnt pathways that were present in our data. In contrast to cell adhesion molecules, longer biotin labelling time increased the detection of known β-catenin interactors in the Wnt signalling pathway (Fig. 3E). We thus conclude that proximity labelling is suitable for the identification of β-catenin interacting proteins, and that the detection of cytoplasmic and nuclear interactors may be boosted by increased biotin labelling time.

Additionally, we assessed the possibility of extending our data by tagging endogenous β-catenin with TurboID. Although we successfully established 293 T clonal cell lines with TurboID-tagged β-catenin (Fig. 1F, G), analysis of mass spectrometry data derived from these cells detected just two significantly enriched proteins after biotin labelling for up to 30 min (DCAF7 and RAB11A; Supplemental Fig. 1, and Supplemental Table 4). More critically, we did not detect the bait protein, β-catenin, in any experiment, likely due to its relatively low abundance in unstimulated cells (Fig. 1F). At this point, it is unclear if experimental modifications, such as the induced stabilisation of cytosolic β-catenin, would improve detection efficiency.

### Comparison of proximity proteomics with affinity purification proteomics data

Several research groups have previously studied the interactome of β-catenin using AP-MS. Of these earlier studies, we selected seven to compare with our proximity proteomics data^6–12^ (Supplemental Table 5). For well-established β-catenin interactors in cell adhesions and the Wnt signalling pathway, we noted that the TurboID results, in particular, compared favourably to AP-MS data (Table 1). Specifically, we detected several components of the cytoplasmic β-catenin destruction complex (e.g., APC, β-TrCP, GSKα/β), the nuclear Wnt enhanceosome^28,29^ (e.g., ICAT, SMARCA4), and cellular junctions, similar to these earlier studies. In contrast, we did not detect any of the four β-catenin-associated TCF/LEF family transcription factors, possibly because some components of larger protein complexes may be occluded from biotin labelling. Of note, this observation is consistent with our earlier study on FOXQ1, which we validated as a bona fide TCF7L2 interactors despite our inability to detect FOXQ1/TCF/LEF interaction by proximity proteomics^30^.

**Table 1:** Discovery of known β-catenin interactors by proximity proteomics and comparison with earlier studies.

		**Proximity proteomics (this study)**							**Affinity purification-based proteomics**						
	**Interactor (gene name)**	**BioID-CTNNB1 18 h rep #1**	**BioID-CTNNB1 18 h rep #2**	**BioID-CTNNB1 18 h rep #3**	**CTNNB1-TurboID 0.5 h**	**CTNNB1-TurboID 3 h**	**TurboID-CTNNB1 0.5 h**	**TurboID-CTNNB1 3 h**	**PMID: 15,492,215**	**PMID: 17,510,365**	**PMID: 24,240,237**	**PMID: 24,201,312**	**PMID: 31,338,436**	**PMID: 30,630,973**	**PMID: 36,315,912**
**β-catenin destruction complex**	APC	x	x	x	x	x	x			x	x			x	x
	AXIN1					x				x	x				x
	AXIN2									x	x			x	x
	BTRC				x	x	x								x
	CSNK1A1									x	x			x	
	FBXW11														x
	GSK3A				x	x	x			x					
	GSK3B					x	x			x				x	
**Wnt enhanceosome**	BCL9	x													x
	BCL9L														x
	CREBBP				x		x								x
	CTNNBIP1	x	x	x	x	x	x	x		x	x			x	
	DVL1														
	DVL2														
	DVL3														
	EP300				x	x								x	x
	LEF1									x	x			x	x
	SMARCA4				x	x	x	x						x	
	TCF7									x				x	x
	TCF7L1														
	TCF7L2										x			x	x
**Cell adhesion**	CDH1										x				
	CDH2	x	x	x	x	x	x	x		x	x	x			x
	CTNNA1	x	x	x	x	x	x	x	x	x	x	x	x	x	x
	CTNND1	x	x	x	x	x	x			x	x		x	x	
	JUP	x	x	x	x	x	x	x		x		x		x	x

x indicates the detection of well-established β-catenin interactors in the individual experiments after subtraction of the corresponding control. Numbers in the proximity proteomics experiments indicate the biotin labelling time. Results from earlier studies are shown as reported by the authors irrespective of any filtration and normalisation. PMID: PubMed identification number.

**Fig. 4:**
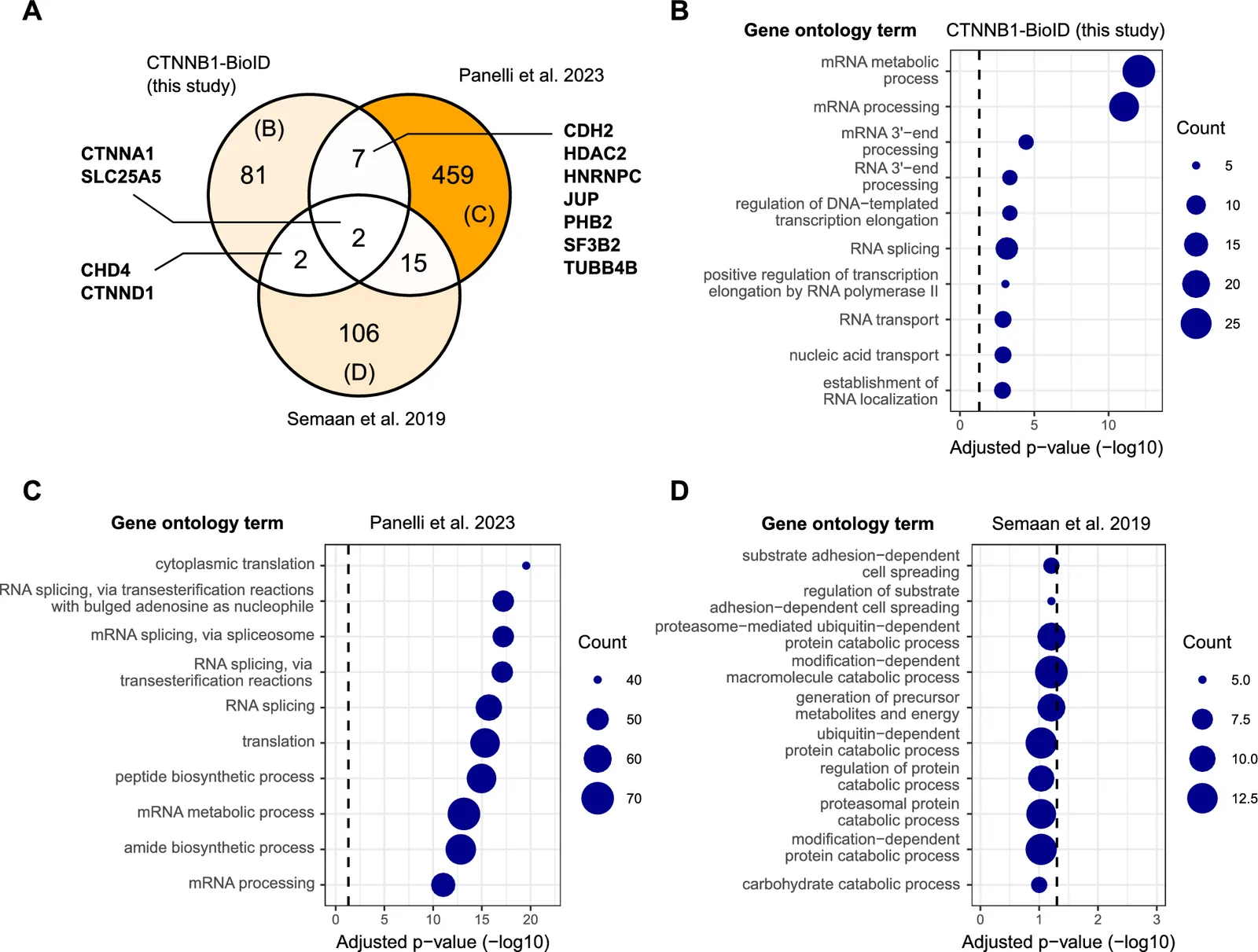
Comparison of BioID results with published affinity purification/mass spectrometry data. (**A**) Venn diagram illustrating the overlap between our BioID data and two recent studies that investigated the interactome of β-catenin using mass spectrometry following affinity purification. For the purpose of this comparison, we included all candidates from our data with an adjusted p-value < 0.05 that were not classified as possible contaminants (n = 92). (**B**-**D**) Gene ontology analysis of candidate β-catenin interactors uniquely identified in the individual studies. The top 10 most significantly enriched GO terms are shown for each dataset. P-values were corrected using the Benjamini–Hochberg procedure.

To further investigate the overlap of our results with previous reports, we focused on two recent studies that employed similar data acquisition and analysis methodology^6,10^, including selection of controls and normalisation strategy, thus allowing for direct comparison. We observed that only few high-confidence interactors were shared between the individual studies, including the cell adhesion molecules α/δ-catenin, N-cadherin, and plakoglobin (Fig. 4A). Gene ontology analysis of uniquely identified interactors suggested that both our experiments and those by Panelli et al.^6^ identified numerous (but discrete) proteins involved in RNA processing (Fig. 4B, C). In contrast, Semaan et al.^10^ primarily identified β-catenin interactors involved in cell spreading and protein catabolism. We conclude that individual proteomics studies discover largely separate sets of interactions, which opens an opportunity for the identification of previously unknown β-catenin interactors by proximity proteomics.

### Proximity proteomics prioritise potential new β-catenin interactors

**Fig. 5:**
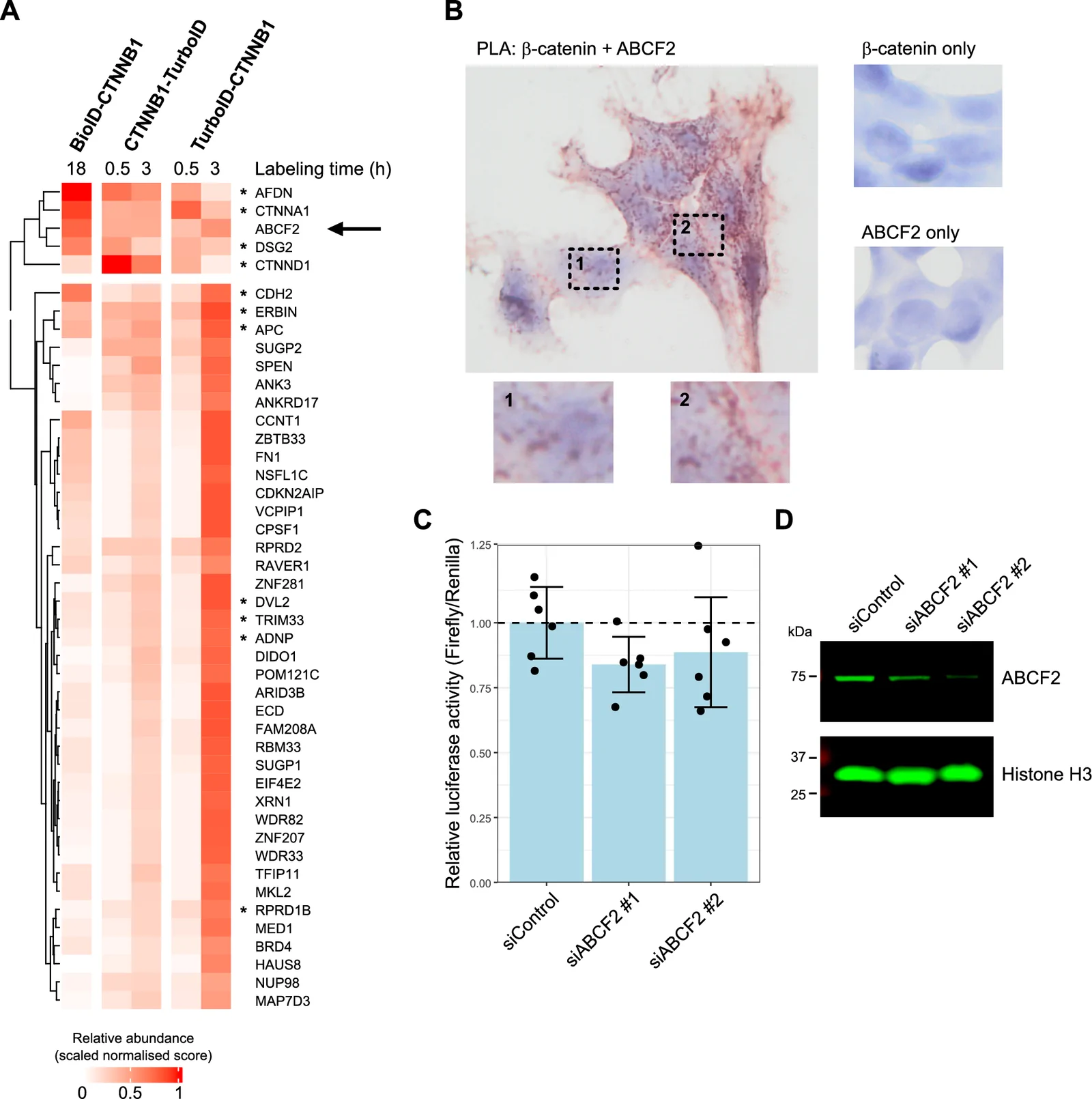
Identification of ABCF2 as a putative new b-catenin interactor. (**A**) Heatmap of candidate β-catenin interactors identified in all proximity proteomics experiments after normalisation for the respective control conditions. Results were grouped and sorted by unsupervised hierarchical clustering. Asterisks indicate previously validated β-catenin interacting proteins. Biotin labelling times are shown above the heatmap. (**B**) Proximity ligation assay (PLA) of β-catenin and ABCF2 in HCT116 cells. Brown dots indicate individual protein–protein interactions. Nuclei are counterstained in blue. Representative areas are shown magnified below the image. Single antibody negative controls are shown in the images to the right. (**C**) TOPflash reporter assay of β-catenin-dependent transcriptional activity in DLD1 cells. (**D**) Immunoblot of ABCF2 levels in HCT116 cells following knock-down with the indicated siRNAs. Histone H3 was used as a loading control.

To determine whether proximity proteomics can aid in the discovery of new β-catenin interactors, we applied unsupervised hierarchical clustering to our combined BioID and TurboID data (Fig. 5A). We observed that the data separated into two distinct clusters. Cluster 1 encompassed proteins that were detected at high levels in all experiments and contained several well-established β-catenin interactors in cell adhesions. Cluster 2, on the other hand, was comprised of proteins whose relative spectral counts increased with biotin labelling time in the TurboID experiments. Out of these proteins, approximately 18% have previously been validated as β-catenin interactors.

We noted that Cluster 1 contained one additional protein, ABCF2, that has not been identified as a β-catenin interactor so far. In a prior genome-wide RNA interference screen of possible Wnt/β-catenin signalling regulators^31^, knock-down of ABCF2 using two separate siRNA pools considerably reduced Wnt pathway activity in DLD1 colorectal cancer cells (Supplemental Fig. 2A), highlighting ABCF2 as a putative positive regulator of Wnt/β-catenin signalling. We did not detect β-catenin/ABCF2 association by regular co-immunoprecipitation in DLD1 cells (Supplemental Fig. 2B), suggesting that the proteins do not form a stable protein complex. However, proximity ligation assays in HCT116 cells indicated abundant and ubiquitous β-catenin/ABCF2 interaction in situ (Fig. 5B). Moreover, RNA interference in DLD1 cells using two independent ABCF2 siRNAs resulted in a consistent (albeit non-significant) reduction in β-catenin transcriptional activity by approximately 20% (Fig. 5C, D). Taken together, these results suggest that ABCF2 may be a β-catenin interacting protein that acts as a positive regulator of Wnt/β-catenin signalling.

## Discussion

Β-catenin is an important regulator of cell adhesion and gene transcription. Because of its critical role in cellular homeostasis, it is of considerable interest to identify β-catenin interactors that mediate its functions in health and disease. In this study, we describe the generation and validation of β-catenin expression constructs for proximity proteomics, and demonstrate the usefulness of these tools for expanding the interactome of β-catenin.

Previous studies have extensively used affinity purification followed by mass spectrometry for the discovery of β-catenin interactors^6–12^. Compared to this experimental approach, BioID/TurboID-based proteomics has several advantages as well as disadvantages that should be considered. Firstly, the main advantage of proximity proteomics is that it does not require protein complexes to stay intact during the cell lysis and protein isolation step. This allows for the detection of interactors that engage the protein-of-interest transiently or with low affinity^13^. Secondly, the relatively short biotin labelling time when using TurboID-based constructs can facilitate the interrogation of protein–protein interactions in specific contexts, e.g., the formation of transcriptional complexes after cell stimulation^14^. On the other hand, protein biotinylation indicates that two proteins are in close proximity (with a labelling radius of approximately 10 nm)^32^, but does not necessarily imply protein interaction, which need to be validated empirically. Accordingly, AP-MS and proximity proteomics can be used to identify discrete yet biologically meaningful sets of interactors of proteins-of-interest^15^.

One point of note is that we were unable to detect numerous known β-catenin interactors, including the TCF/LEF transcription factors or other nuclear components of the β-catenin transcriptional complex, using proximity proteomics. Several possible explanations for this seem possible. On the one hand, the addition of a large functional domain may interfere with engagement of β-catenin with the transcriptional complex. However, our constructs retained at least partial activity in Wnt reporter assays, arguing against complete loss-of-function. On the other hand, TCF/LEF proteins may have low abundance or solubility, making them difficult to detect by mass spectrometry (see Table 1).

Despite these limitations, our data highlight several candidate β-catenin interactors that will be of considerable interest for further validation. Most notably, we detected the atypical ABC family transporter ABCF2 across all assays. ABCF2 is a ubiquitous yet poorly characterized protein that, in contrast to other ABC family members, lacks a transmembrane domain and localises to cytosol and nucleus^33^. ABCF2 has been implicated in the pathogenesis of multiple types of cancer, including Wnt/β-catenin-driven cancers such as ovarian and breast cancer^34,35^. An earlier genome-wide RNA interference screen in DLD1 colorectal cancer cells identified ABCF2 as a potential positive regulator of Wnt/β-catenin signalling^31^, which was consistent with our experimental results. Although we were unable to detect β-catenin/ABCF2 interaction by co-immunoprecipitation, we observed strong association of the two proteins in proximity ligation assays that do not require stable protein–protein interaction. ABCF2 was also detected as a possible β-catenin interactor in an AP-MS dataset from K562 myelogenous leukaemia cells^7^, a Wnt-dependent cell line with relative high β-catenin protein levels, which supports the notion that ABCF2 is a *bona fide* β-catenin interacting protein. We thus hypothesise that β-catenin and ABCF2 form transient complexes, which are known to be important for various biological processes^36^. Of note, both ABCF2 and β-catenin have independently been shown to bind to the actin-binding protein actinin-4^37,38^, suggesting that the two proteins form part of a larger, possibly cytoskeleton-associated complex. Alternatively, ABCF2 has been shown to associate with ANKRD6^31^, a known negative regulator of Wnt/β-catenin signalling that acts on the level of the β-catenin destruction complex^39^, and might thereby boost Wnt signalling.

In summary, we propose proximity proteomics as an important complementary tool for the study of β-catenin biology. We anticipate that this experimental approach will facilitate the discovery of functional β-catenin interactors that have eluded detection in earlier studies, since affinity purification depends on stable protein–protein interaction. For example, our data highlight several potential interactors involved in RNA processing and transcription regulation such as RAVER1, SUGP1/2, and KAISO/ZBTB33, which could feasibly contribute to β-catenin-dependent gene regulation. Thus, the molecular tools and data presented here may aid in the identification of new therapeutic targets for β-catenin-dependent cancers.

## Supplementary Information


Supplementary Information 1.
Supplementary Information 2.


## Data Availability

The mass spectrometry data have been deposited as a complete submission to the ProteomeXchange Consortium via the PRIDE^40^ partner repository with identifier PXD067416.
