# Supplementary material for: Comprehensive proximity proteomics expand the known interactome of the oncoprotein β-catenin
Source: Sci Rep. 2026 Sep 1;16:27374. doi: 10.1038/s41598-026-68008-5 (PMC13534447; doi:10.1038/s41598-026-68008-5)
Supplement: Supplementary file 1 [file 41598_2026_68008_MOESM1_ESM.pdf]

Supplemental Information

Comprehensive proximity proteomics expand the known interactome of the oncoprotein β-catenin

Lavanya Moparthi[1,2,*], Wenjing Zhong[1,2], and Stefan Koch[1,2,*]

[1]Department of Biomedical and Clinical Sciences (BKV), Linköping University, Linköping, Sweden

[2]Wallenberg Centre for Molecular Medicine (WCMM), Linköping University, Linköping, Sweden

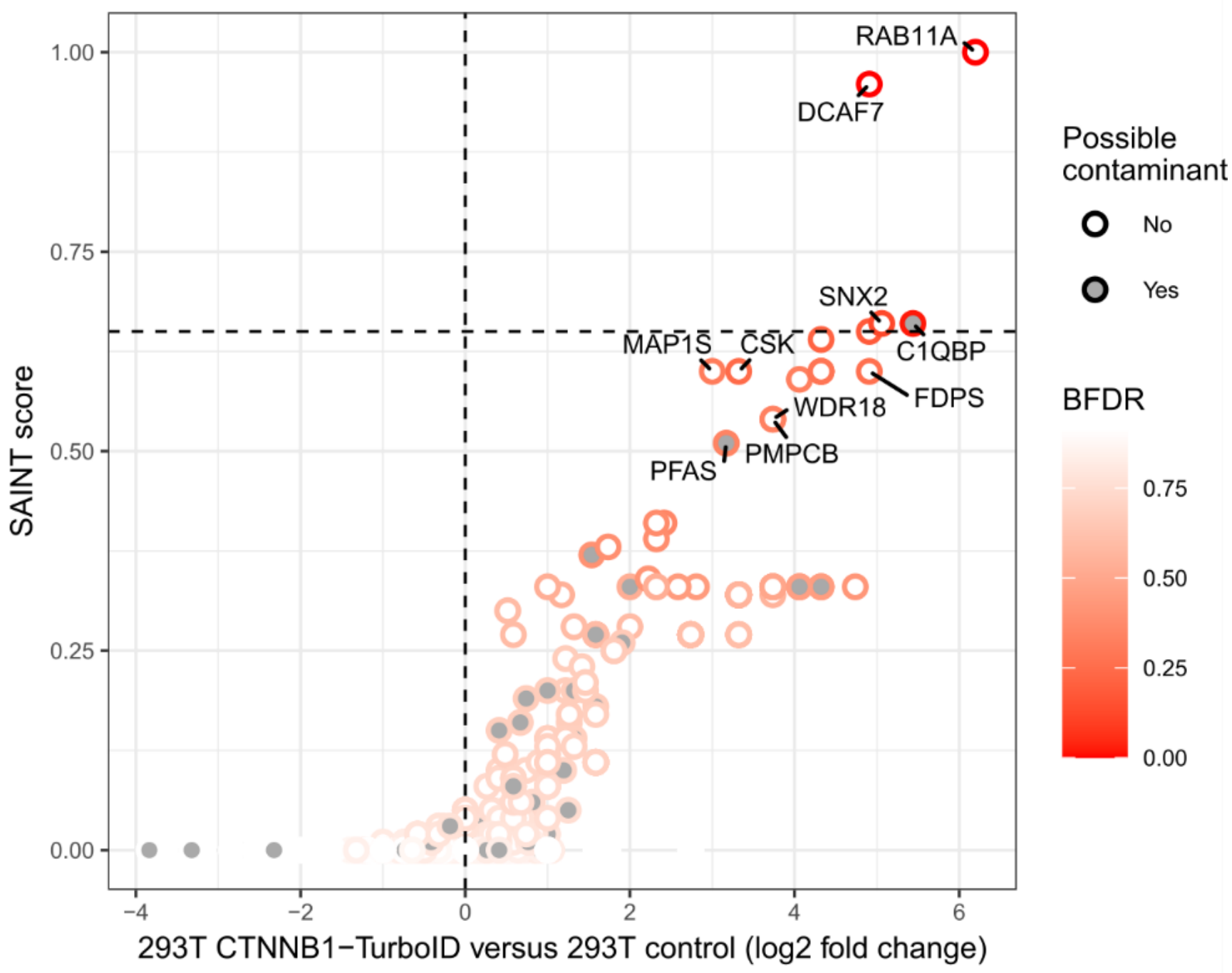

Supplemental figure 1: Exploration of β-catenin interactors using endogenously tagged CTNNB1-TurboID. SAINTexpress analysis of mass spectrometry data derived from 293T cells expressing endogenously tagged CTNNB1-TurboID (see figure 1F, and supplemental table 4). Gene edited and control cells were treated with biotin for 10min, 20min, or 30min (once each). Results from all time points were pooled for the purpose of this analysis. Possible contaminants were defined as having a detection rate ≥ 0.2 and average spectral count ≥ 3 in the CRAPome repository. BFDR: Bayes false discovery rate.

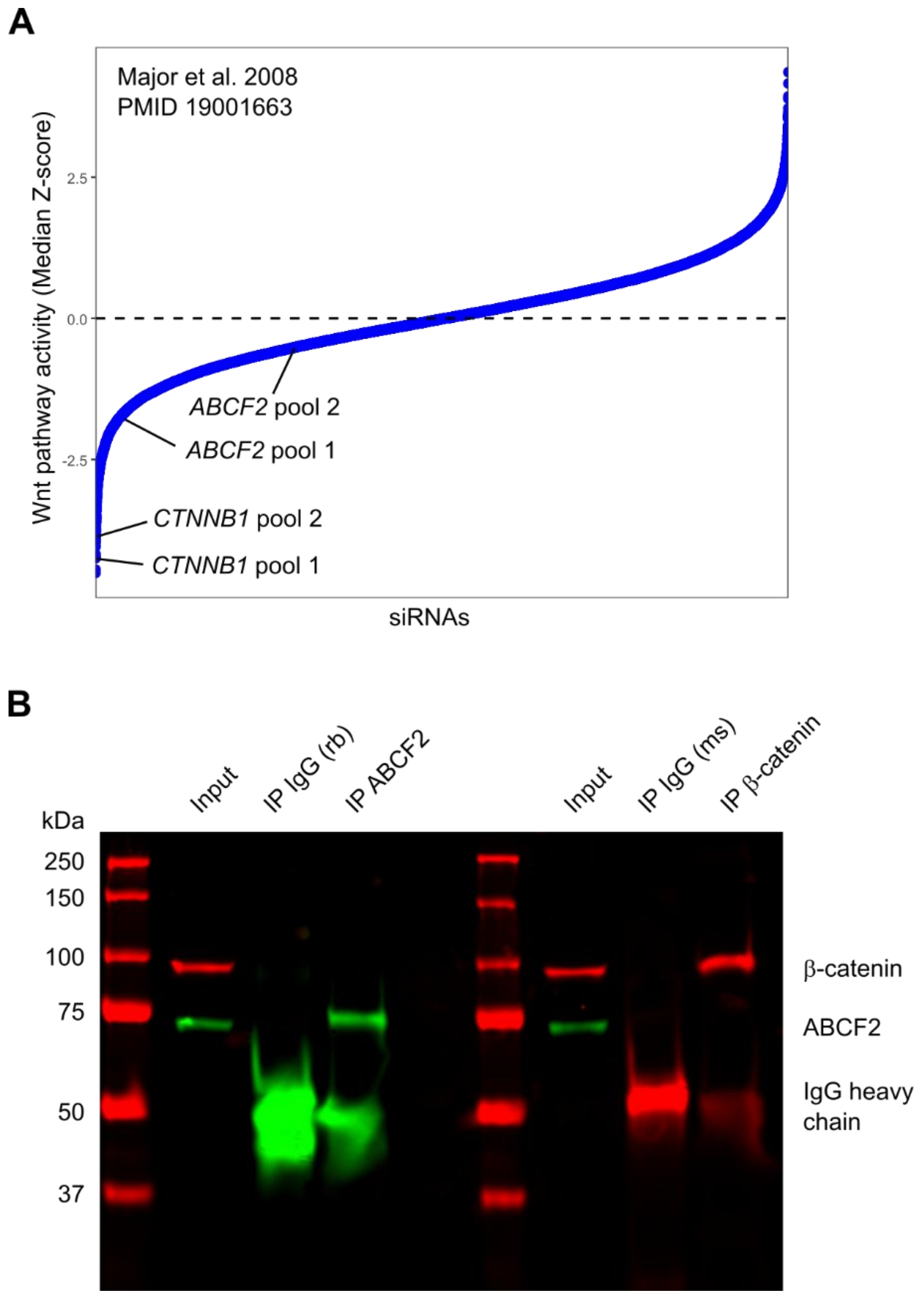

Supplemental figure 2: ABCF2 is a putative regulator of Wnt/β-catenin signalling. (**A**) Re-analysis of a previous genome-wide siRNA screen in DLD1 cells. Two independent siRNA pools targeting ABCF2 reduced Wnt pathway activity. (**B**) Immunoprecipitation of endogenous β-catenin and ABCF2 in DLD1 cells. No co-precipitation was detected in this assay.

Fig. 1A

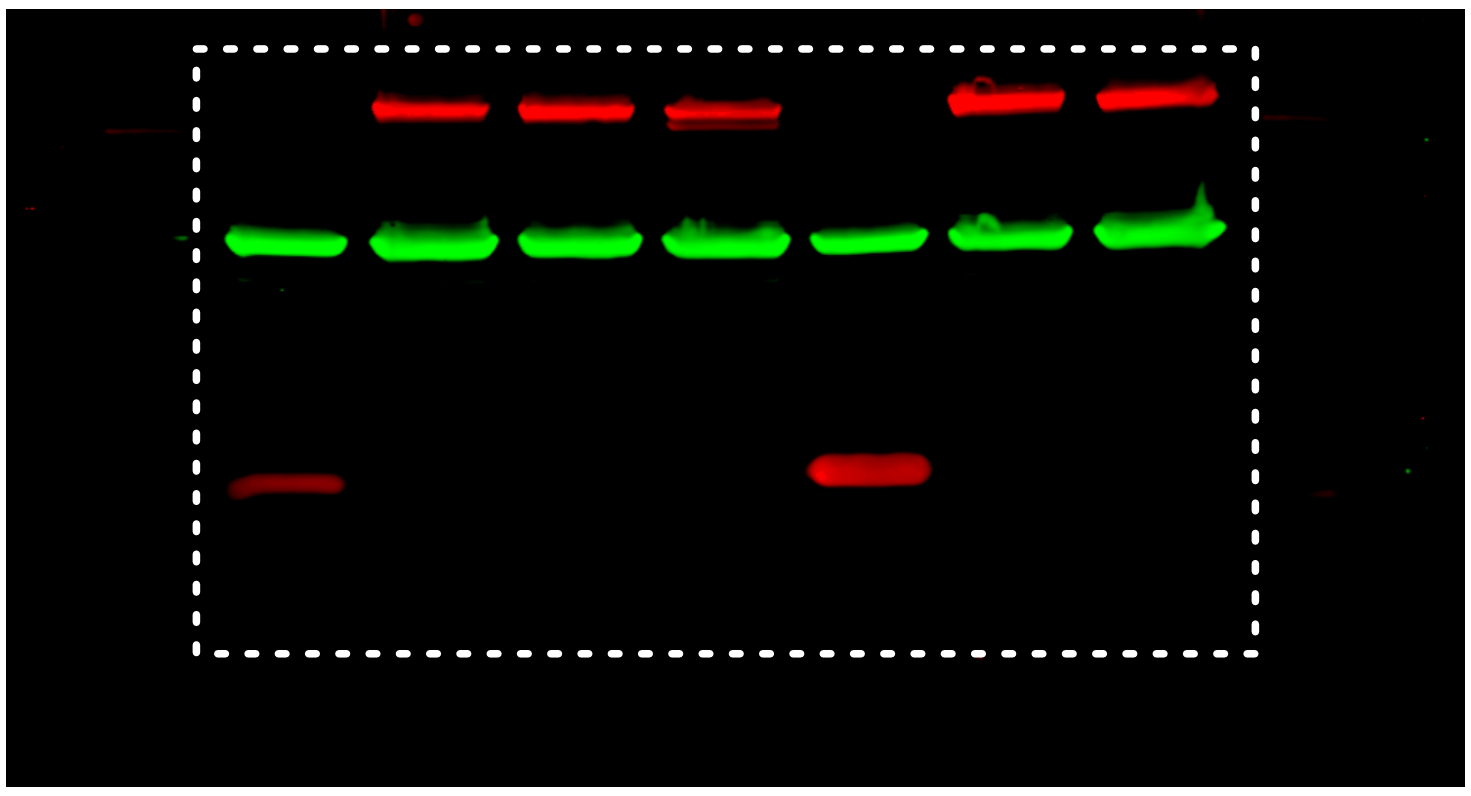

Fig. 1B

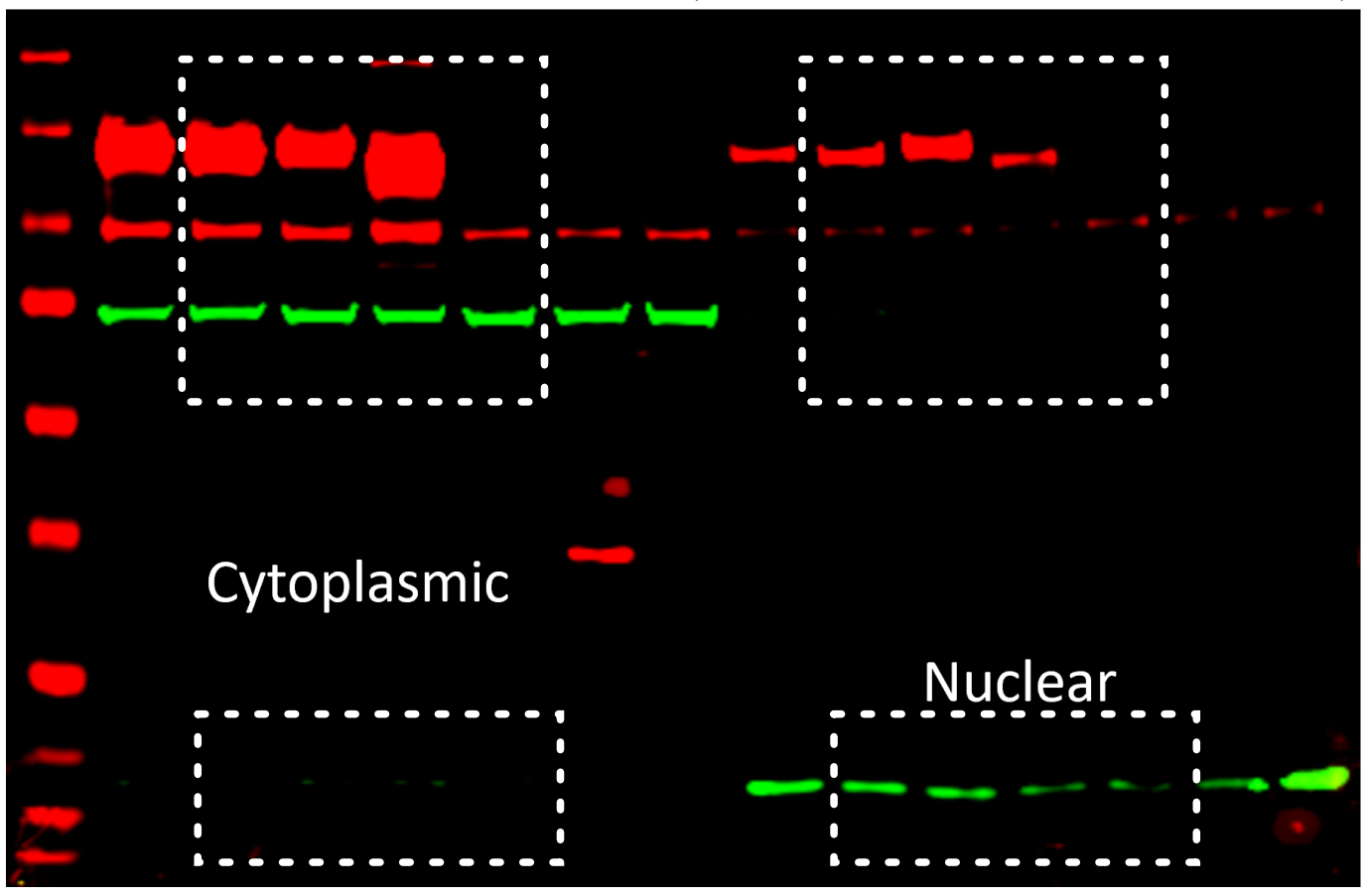

Fig. 1C - left panel

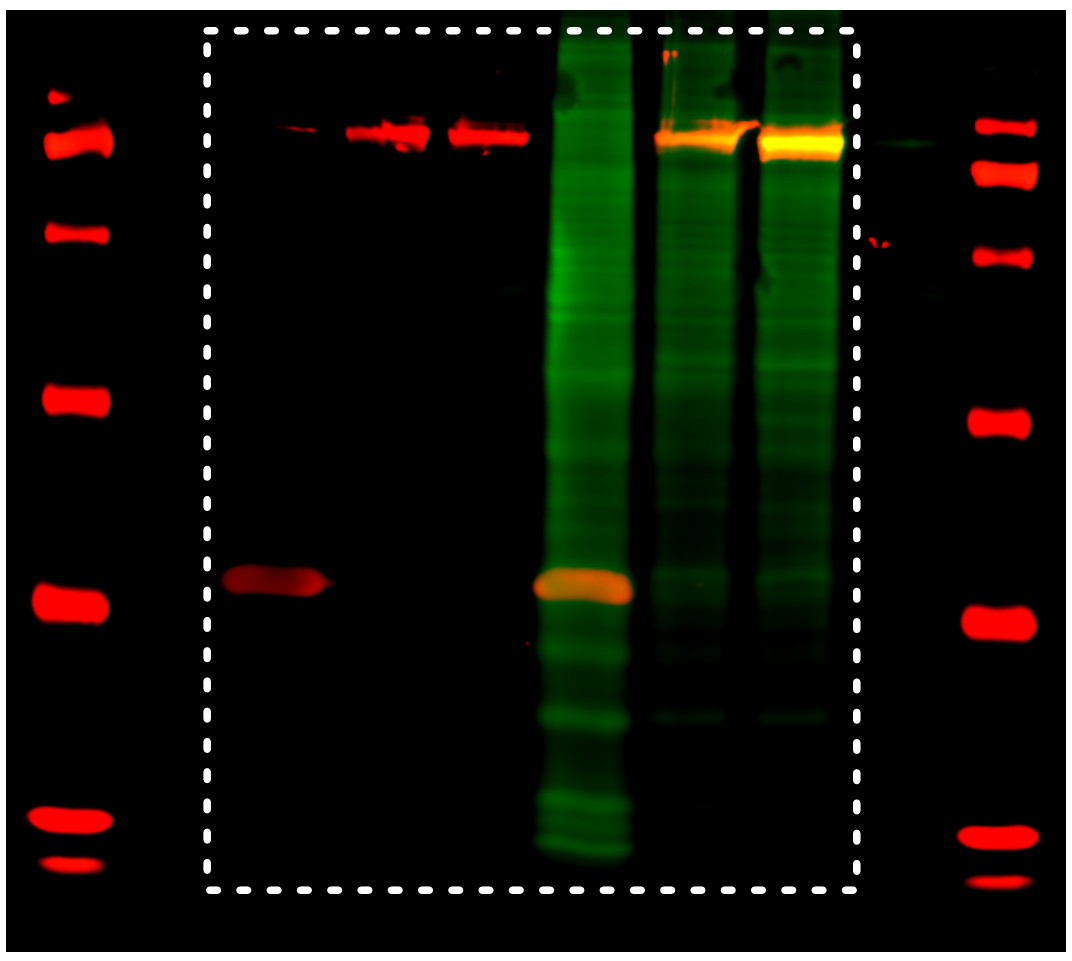

Fig. 1C - right panel

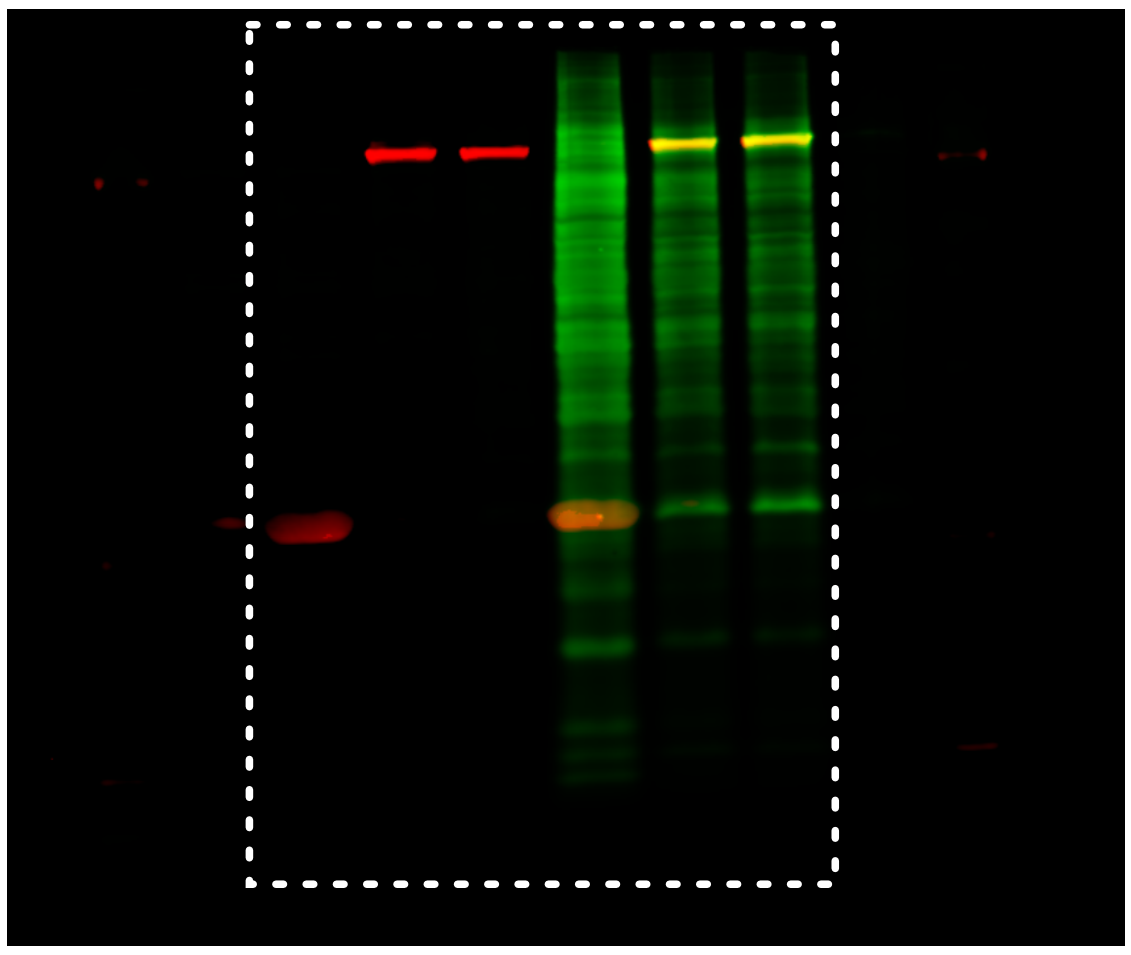

Supplemental figure 3: Uncropped immunoblot images. Dashed boxes idicate the blot areas shown in the manuscript.

Fig. 1F

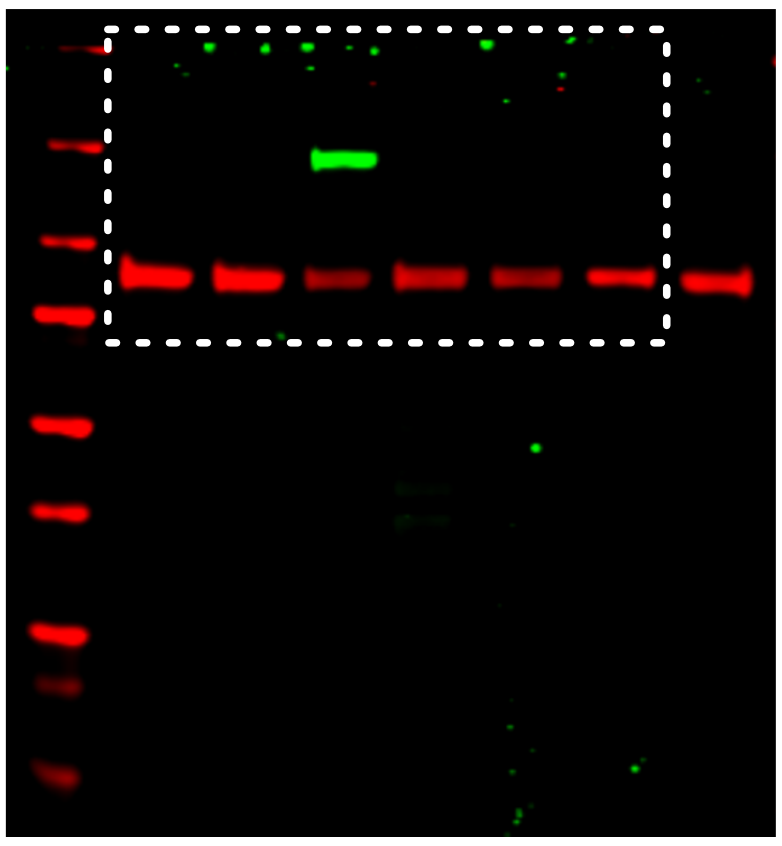

Fig. 5D - upper panel

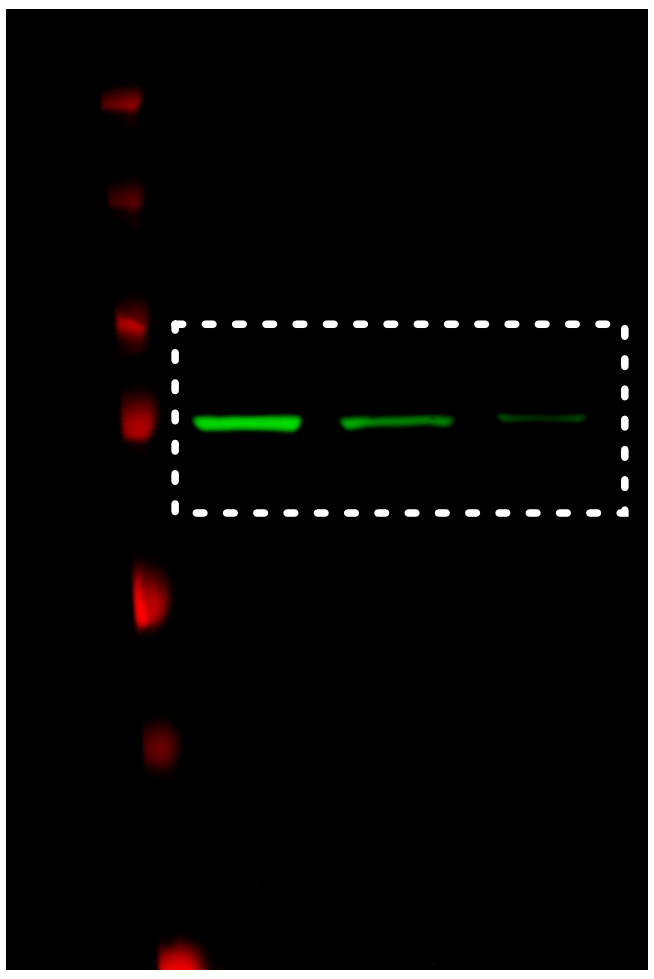

Fig. 5D - lower panel

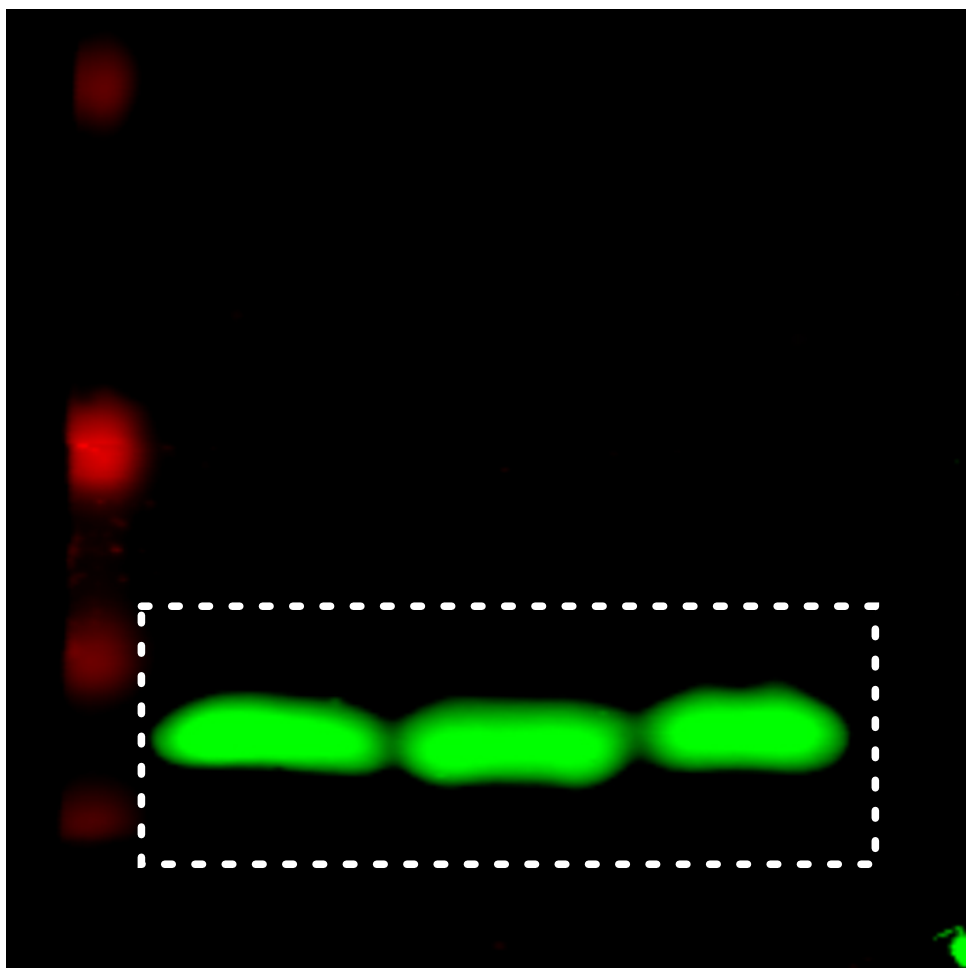

Fig. S2B

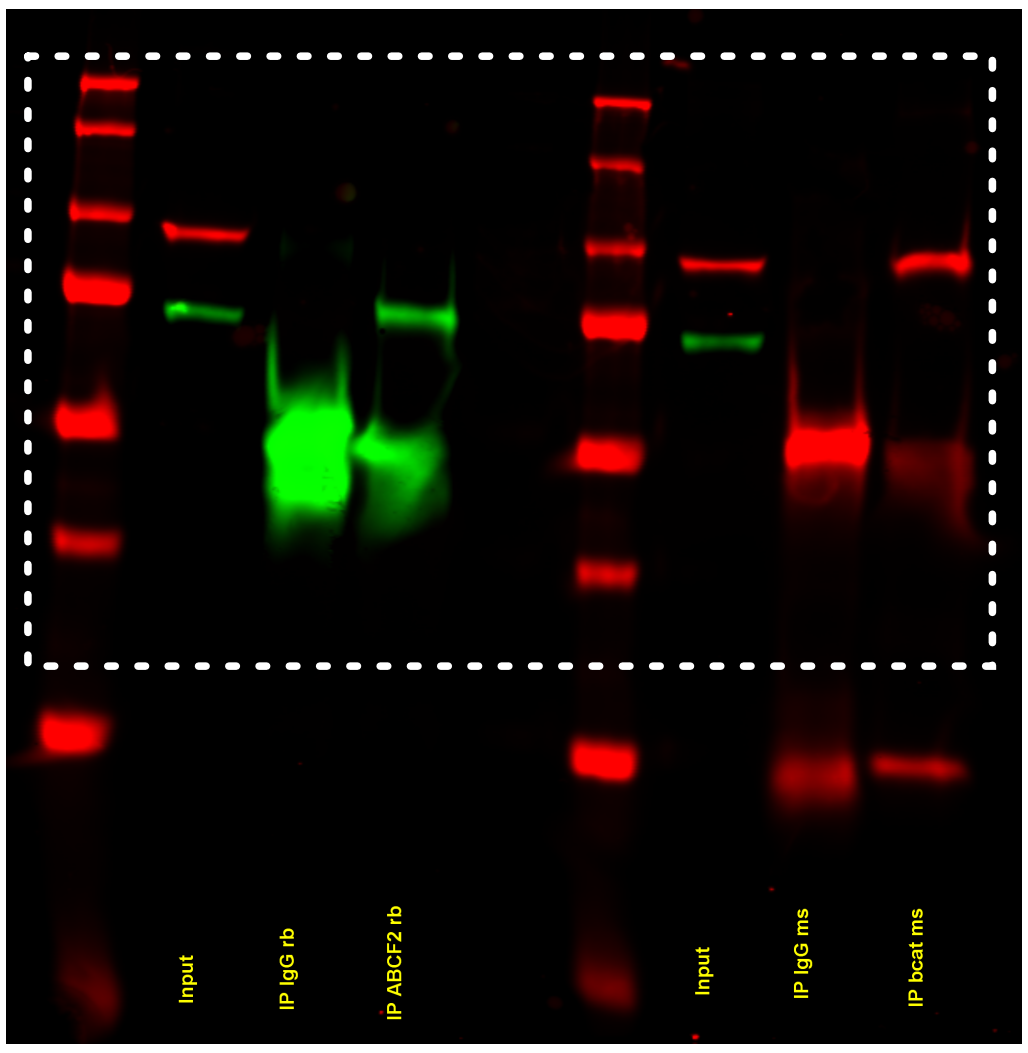

Supplemental figure 3 - continued.
